# Light-induced structural changes in a full-length cyanobacterial phytochrome probed by time-resolved X-ray scattering

**DOI:** 10.1038/s42003-018-0242-0

**Published:** 2019-01-03

**Authors:** Derren J. Heyes, Samantha J. O. Hardman, Martin N. Pedersen, Joyce Woodhouse, Eugenio De La Mora, Michael Wulff, Martin Weik, Marco Cammarata, Nigel S. Scrutton, Giorgio Schirò

**Affiliations:** 1 Manchester Institute of Biotechnology, University of Manchester, 131 Princess St, Manchester, M1 7DN UK; 2 European Synchrotron Radiation Facility, 71 Avenue des Martyrs, 38044 Grenoble, France; 3 Institut de Biologie Structurale, CNRS, Univ. Grenoble Alpes, CEA, 71 Avenue des Martyrs, 38044 Grenoble, France; 4 Univ. Rennes 1, CNRS, UBL, Institut de Physique de Rennes (IPR) - UMR 6251, 263 avenue du Général Leclerc, 35042 Rennes, France

## Abstract

Phytochromes are photoreceptor proteins that transmit a light signal from a photosensory region to an output domain. Photoconversion involves protein conformational changes whose nature is not fully understood. Here, we use time-resolved X-ray scattering and optical spectroscopy to study the kinetics of structural changes in a full-length cyanobacterial phytochrome and in a truncated form with no output domain. X-ray and spectroscopic signals on the µs/ms timescale are largely independent of the presence of the output domain. On longer time-scales, large differences between the full-length and truncated proteins indicate the timeframe during which the structural transition is transmitted from the photosensory region to the output domain and represent a large quaternary motion. The suggested independence of the photosensory-region dynamics on the µs/ms timescale defines a time window in which the photoreaction can be characterized (e.g. for optogenetic design) independently of the nature of the engineered output domain.

## Abstract (web summary)

Heyes et al. report that the conformational changes of a full-length *Synechocystis* phytochrome from the red- to the far-red-absorbing states extend to the seconds timescale. Interestingly, while the rapid structural changes are independent of the output domain the slower changes are not, providing useful insights on photosensory-region dynamics.

## Introduction

Phytochromes are light-sensing proteins that are able to transduce a light signal into a biochemical output in plants, bacteria and fungi^1,2^. The photosensory activity of phytochromes results from their capacity to undergo a light-induced and reversible switching between two conformers, a red-light-absorbing Pr form and a far-red-light-absorbing Pfr form, which are characterized by distinct 3D structures and spectral properties. Phytochromes are generally soluble and dimeric proteins, with each monomer consisting of multiple domains. The protein consists of an N-terminal photosensory region that comprises three domains, namely a PAS, GAF, and PHY domain, and a C-terminal output domain, which is normally a histidine kinase domain. The light-sensing properties of phytochromes result from the presence of a bilin chromophore, either phytochromobilin in plant phytochromes, phycocyanobilin in cyanobacterial phytochromes or biliverdin in bacterial phytochromes, which is covalently attached to a conserved cysteine residue within the GAF domain. Due to their near-infrared spectral sensitivity, where the absorbance of other biological macromolecules is low, the phytochrome family is becoming an important target for optogenetic applications and they are the preferred templates for designing optical molecular tools for applications in mammals^3^.

The mechanism by which phytochromes transmit the light signal from the chromophore through the photosensory module, and ultimately into the regulatory module to actuate signaling remains unclear. For most phytochromes, there is a general consensus that the absorption of a photon by the chromophore in the Pr form triggers a *Z/E* isomerization of the C15-C16 double bond between the C and D rings of the tetrapyrrole on the picosecond timescale^4,5^ accompanied by deprotonation/reprotonation of the pyrrole nitrogens^6^. Isomerization induces rotation of the D ring, which is followed by a translation of the bilin chromophore within its binding pocket^5^. Photoisomerization of the chromophore is then followed by slower steps on the micro- to millisecond timescale, which have been proposed to involve global changes in protein structure, to form an active signaling conformation of the C-terminal region in the final Pfr state^7,8^. A recent comparison between the crystal structures of a dark-adapted form and a red-light illuminated form has provided a model for the structural changes in the photosensory module of a bacterial phytochrome^9^. It was proposed that a unique tongue region, extending from the chromophore binding domain to the adjacent PHY domain, undergoes a change in secondary structure from an anti-parallel β-sheet configuration in the Pr state to an α-helix in the Pfr state^9,10^. This change in secondary structure is thought to induce a strain along the dimer interface to force the opening of the neighboring PHY domains. Although this motion has only been observed in crystals of the photosensory region of a phytochrome^9^, it provides a plausible mechanism to transmit the light signal to the output domain in the full-length protein via a structural motion that triggers downstream signaling. While the initial *Z*/*E* photoisomerization of the bilin chromophore at the C15-C16 position has been confirmed by several studies, it is still largely unknown how isomerization alters the bilin conformation, changes the structure of the bilin binding pocket, and ultimately triggers a rearrangement of the interface between the chromophore binding domain and the adjacent domains that results in the secondary structure change in the tongue region. It is also unclear if these motions that have been observed in the photosensory domain^8–10^ are affected by the presence of the output domain in the full-length phytochrome.

In order to fully exploit phytochromes as optogenetic tools it will be necessary to structurally reassemble the natural output domains with other biologically-relevant output modules^3^. This rational design process will ultimately require a detailed spatio-temporal understanding of the structural changes that occur in full-length phytochrome proteins. However, no 3D structure of a full-length phytochrome containing an output domain has been solved yet and it is unknown how structural changes in the photosensory module extend to the C-terminal regulatory module to trigger the signaling process. Time-resolved X-ray scattering studies remain the most attractive approach for tracking structural transitions of phytochromes in solution that follow the initial photochemical event. Recent time-resolved X-ray scattering studies have described the structural dynamics of a bacterial biliverdin-containing phytochrome in both the photosensory region on its own and in a full-length form^8,9,11^. The major structural events in the bacterial phytochrome were found to occur from tens of microseconds to a few milliseconds in both forms of the protein. It was proposed that the entire phytochrome protein undergoes a concerted structural rearrangement, resulting in the twisting of the output domains prior to the formation of the final Pfr state^8^. However, it is unclear if the structural changes are specific for the bacterial homologs or if the mechanism is conserved across the plant and cyanobacterial phytochromes, which undergo more complex reaction cycles.

**Fig. 1:**
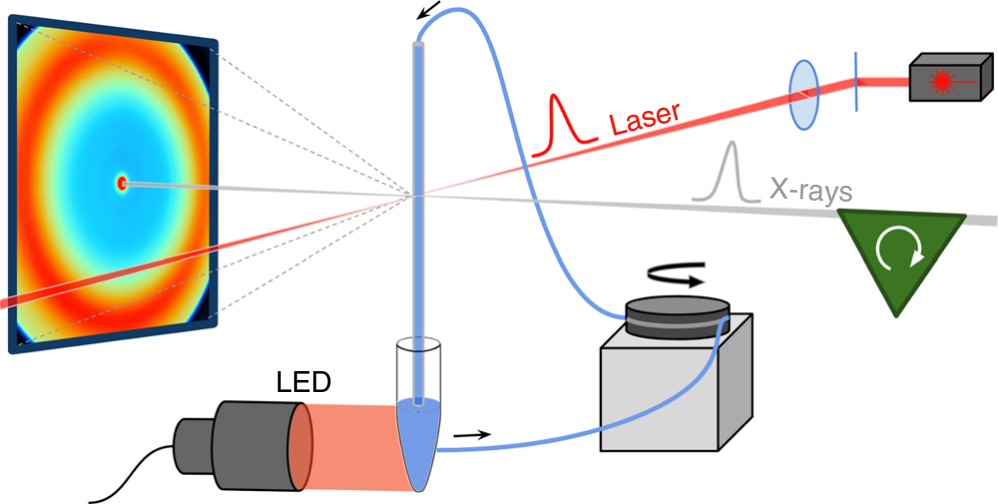
Experimental setup. Schematic representation of the experimental setup for static and time-resolved S/WAXS experiments at the ID09 beamline of the European Synchrotron Radiation Facility. A protein solution flows through a quartz capillary connected to a peristaltic pump by a teflon loop. Sample reservoir is under continuous illumination by a LED source to stabilize Pr (illumination at 730 nm) or Pfr forms (illumination at 625 nm) for static measurements or to switch back the protein (illumination at 730 nm) to the Pr form in time-resolved experiments to study the Pr to Pfr transition. A nanosecond laser pulse (red) synchronized with a microsecond X-ray pulse train (gray) selected by the ID09 chopper system (green) is used to trigger Pr to Pfr photoconversion and subsequent protein structural changes give raise to changes in the the X-ray scattering pattern measured on a CCD detector (blue)

Here we use a combination of steady-state^12^ and time-resolved^13^ small- $( { \lesssim 0.2\,{\mathrm{{\AA}}}^{ - 1}})$ and wide-angle $( { \gtrsim 0.2\,{\mathrm{{\AA}}}^{ - 1}})$ X-ray scattering (TR-S/WAXS) approaches (Fig. 1) to study the conformational changes that accompany the photoconversion of the Pr state to the Pfr state in a full-length cyanobacterial phytochrome (Cph1) from *Synechocystis* sp. PCC6803. We detect changes in the scattering patterns from the small-angle region up to about 0.7 Å^−1^, and from the microsecond timescale to hundreds of milliseconds, where the signal is still evolving, in agreement with spectroscopic data. The results indicate that the kinetics of structural changes in the full-length cyanobacterial phytochrome extends to the seconds timescale with at least two identifiable intermediates and is more complex than that recently reported for a similar phytochrome from *Deinococcus radiodurans*, where the global structural change occurs in a single concerted motion within a few milliseconds^8^. Moreover, a comparison of TR-S/WAXS and spectroscopic data on the full-length protein with data collected for the photosensory region alone suggests that both localized and global changes on the µs/ms timescale are largely independent of the presence of the output domain, while those at longer times differ significantly in the presence of the output domain. Low-resolution modeling of the static small-angle data suggests that the differences in the small-angle scattering reflect a large motion of the output domains, allowing us to propose a model for the overall structural changes in the full-length cyanobacterial phytochrome.

## Results

### Time-resolved X-ray scattering

**Fig. 2:**
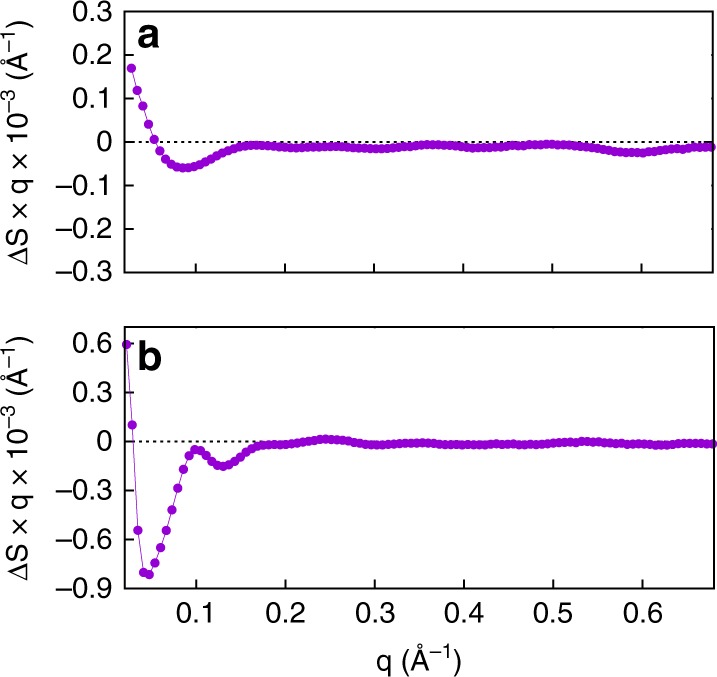
Static X-ray scattering. Static X-ray scattering difference pattern between the Pr and Pfr forms of the photosensory region (**a**) and full-length Cph1 protein (**b**). The difference patterns were calculated by subtracting the X-ray scattering data of the samples illuminated at 625 nm from those illuminated at 730 nm

Time-resolved S/WAXS measurements^13^ were carried out to study the kinetics of the structural transitions during the Pr to Pfr photoconversion for both the full-length Cph1 protein and the photosensory region (PAS-GAF-PHY) of Cph1 upon laser excitation at 630 nm. The experimental setup is shown schematically in Fig. 1 and was initially used to collect the static X-ray scattering difference (Pfr minus Pr) patterns under prolonged illumination with 625 and 730 nm LED light for each protein (Fig. 2). A comparison of the shape of the static difference X-ray scattering pattern of the photosensory region and full-length Cph1 protein reveals major differences in both shape and intensity. The main difference between the photosensory region and the full-length protein can be observed in the small-angle region (q region $\lesssim 0.2\,$Å^−1^) (Fig. 2).

**Fig. 3:**
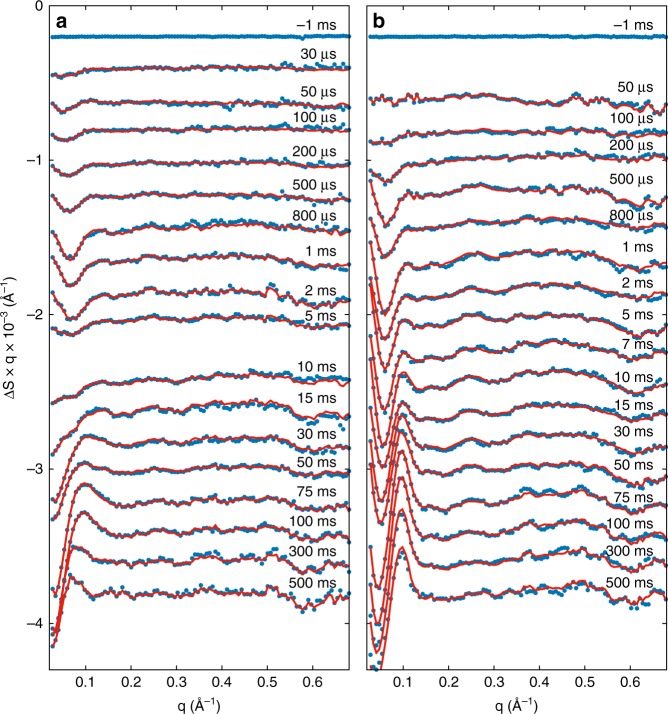
Time-resolved X-ray scattering. Light-induced time-resolved X-ray scattering difference patterns for the photosensory region of Cph1 (**a**) and the full-length Cph1 protein (**b**) in solution. Blue points are experimental data and red lines are linear combinations of the first three basis patterns obtained by singular value decomposition. Data have been vertically offset for clarity. Note that scattering difference patterns are multiplied by the scattering vector q to reduce differences in the amplitude scale in SAXS (≲ 0.2 Å^−1^) and WAXS (≳ 0.2 Å^−1^) regions^12^

**Fig. 4:**
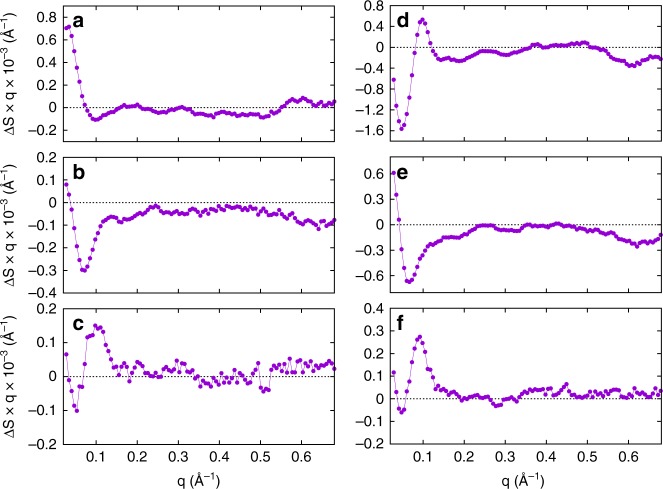
Singular value decomposition analysis. First, second and third basis pattern obtained by singular value decomposition of the entire time-resolved X-ray scattering dataset for the photosensory region (**a**–**c**) and full-length Cph1 protein (**d**–**f**)

**Fig. 5:**
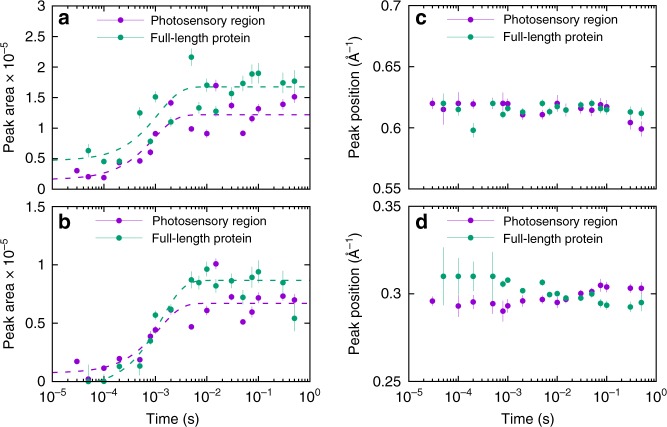
Time evolution of WAXS features. Time evolution of the peak intensity at ~0.62 Å^−1^ (**a**) and ~0.3 Å^−1^ (**b**) for the photosensory region and full-length Cph1 protein. Dashed lines show the fitting in terms of a single exponential with τ_0.62_ = 0.93 ms (photosensory region) and 1.14 ms (full-length), and with τ_0.3_ = 1.18 ms (photosensory region) and 1.30 ms (full-length). The time evolution of the peak positions at ~0.62 Å^−1^ (**c**) and ~0.3 Å^−1^ (**d**) are shown for the photosensory region and full-length Cph1 protein

Time-resolved S/WAXS difference data shows that the signal develops in both intensity and shape on the microsecond-to-second timescale (Fig. 3). Only small changes could be observed on the microsecond timescale for both proteins whereas much more substantial changes in the scattering patterns were obtained on the millisecond timescale. The time-resolved scattering data were analyzed by singular value decomposition, which showed that the entire datasets for both proteins could be described by three main time-independent basis patterns (Figs. 3 and 4). A global kinetic analysis of the whole dataset in terms of the three main basis patterns revealed that the time evolution for both proteins could be fitted to three exponentials with derived lifetimes of τ_1_ = 0.33 ± 0.04 ms, τ_2_ = 41.8 ± 1.5 ms, and τ_3_ = 1540 ± 80 ms for the photosensory region and τ_1_ = 1.69 ± 0.08 ms, τ_2_ = 31.3 ± 2.0 ms, and τ_3_ = 1240 ± 60 ms for the full-length protein. The kinetics of the intensity and the position of the two main features observed in the WAXS region of the time-resolved difference patterns, namely the two negative peaks around 0.62 and 0.3 Å^−1^, are almost identical in data from the photosensory region and the full-length protein (Fig. 5). The time evolution of the intensities of these peaks could be fitted to a single exponential with lifetimes of τ_0.62_ = 0.93 ± 0.40 ms and τ_0.3_ = 1.18 ± 0.5 ms for the photosensory region and τ_0.62_ = 1.14 ± 0.60 ms and τ_0.3_ = 1.30 ± 0.24 ms for full-length Cph1 (Fig. 5a, b), which are similar to the first lifetime obtained from the global kinetic analysis (0.33 ± 0.04 ms for the photosensory region and 1.69 ± 0.08 ms the full-length protein). However, the position of these peaks does not change over time (Fig. 5c, d).

**Fig. 6:**
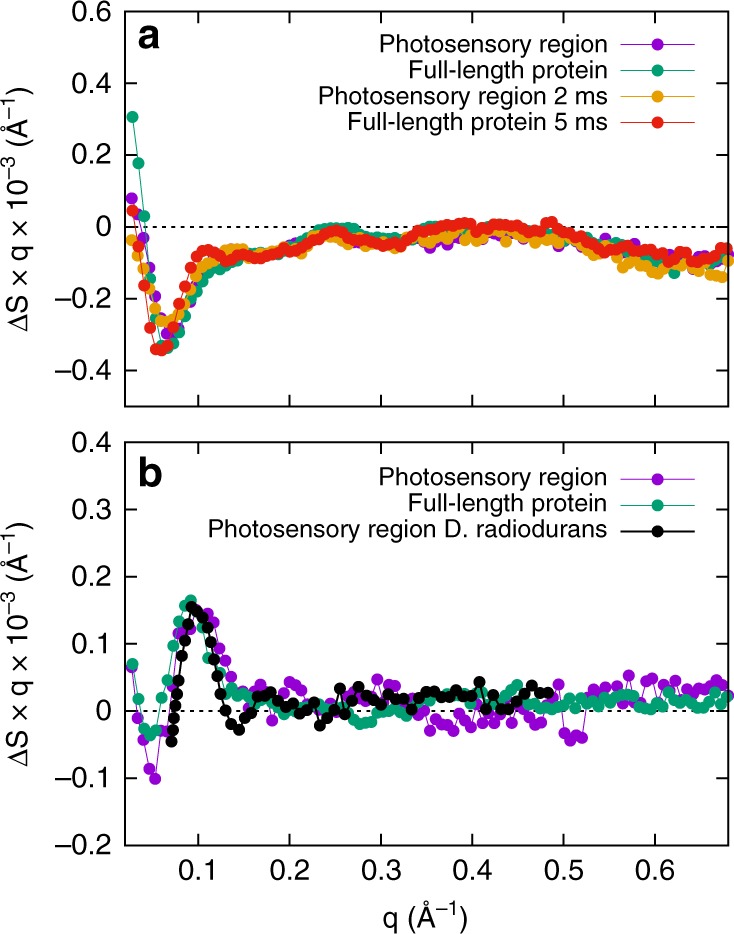
Interpretation of basis patterns. **a** A comparison of the second basis patterns obtained by singular value decomposition of the photosensory region and full-length Cph1 protein with the transient experimental signals at 2 and 5 ms, respectively. **b** A comparison of the third basis patterns obtained by singular value decomposition for the photosensory region and full-length Cph1 protein with the experimental transient pattern measured on the photosensory module of a bacterial phytochrome from *Deinococcus radiodurans*^9^

A direct comparison of the basis patterns obtained for both forms of the protein provides further insights into the role of the output domain in the structural rearrangements (Fig. 6). The second and third basis patterns, which represent the signal developing on the micro-to-millisecond timescale, are very similar between the full-length and truncated forms of the protein. This is confirmed by a comparison of the shape of the X-ray difference signal after a few ms and the second basis patterns, which appear to be nearly identical for both the photosensory region and the full-length Cph1 protein (Fig. 6a). The third basis patterns for both proteins are also similar to the experimental difference pattern observed for the photosensory module of the bacterial phytochrome from *Deinococcus radiodurans* upon Pr-to-Pfr photoconversion (Fig. 6b)^9^. However, there are considerable differences between the photosensory region and the full-length protein in both the shape and intensity of the first basis pattern, which represents the signal that is formed on the timescale of hundreds of milliseconds (compare panels a and d in Fig. 4). It should be noted that for both proteins the transient X-ray scattering signal at the longest time-delay measured (500 ms) has not yet fully developed the shape of the static Pfr–Pr difference pattern (Figs. 3 and 4), but it already contains a major component of the final signal, as shown by the close similarity of the first basis patterns (Fig. 4a, d) with the static difference signal (Fig. 2).

### Transient absorption spectroscopy

**Fig. 7:**
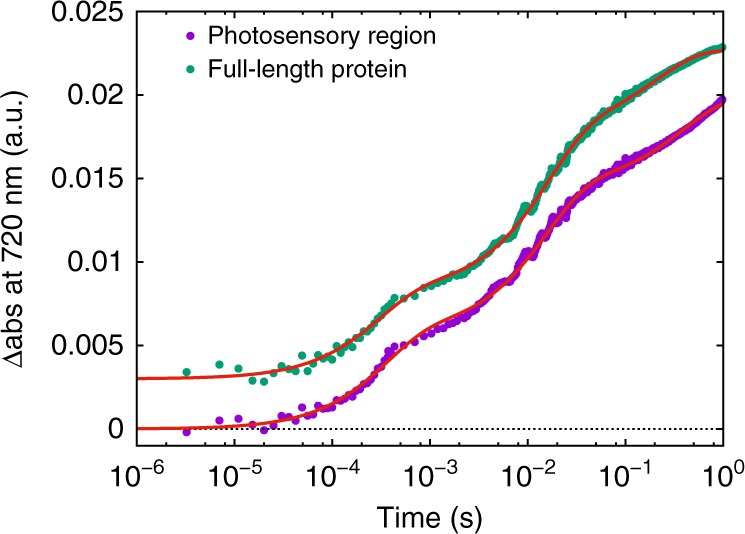
Time-resolved spectroscopy. Time evolution of the absorption change at 720 nm after laser excitation at 630 nm for the photosensory region of Cph1 (purple points) and the full-length Cph1 protein (green points). Red lines are fitting in terms of a combination of three single exponentials with τ_1_ = 0.345 ms, τ_2_ = 15.0 ms, τ_3_ = 393 ms (photosensory region) and with τ_1_ = 0.294 ms, τ_2_ = 15.6 ms, τ_3_ = 209 ms (full-length protein). Data and fitting curve of full-length protein have been vertically offset by 0.003

In order to correlate the temporal behavior of the structural changes with optical changes, transient absorption spectroscopy measurements were performed on the full-length Cph1 protein and the photosensory region (PAS-GAF-PHY) of Cph1 on the microsecond-to-second timescale. Kinetic transients were recorded at 720 nm following excitation with a nanosecond laser pulse at 630 nm to report on the formation of the Pfr state (Fig. 7). The data were fitted to a combination of three exponentials with derived lifetimes (τ_1_ = 0.345 ± 0.010 ms, τ_2_ = 15.0 ± 0.2 ms, τ_3_ = 393 ± 11 ms for the photosensory region and τ_1_ = 0.294 ± 0.009 ms, τ_2_ = 15.6 ± 0.2 ms, τ_3_ = 209 ± 4 ms for the full-length protein) that are similar to those reported previously on the same protein^7,14,15^.

### Ab initio modeling of static SAXS data

**Fig. 8:**
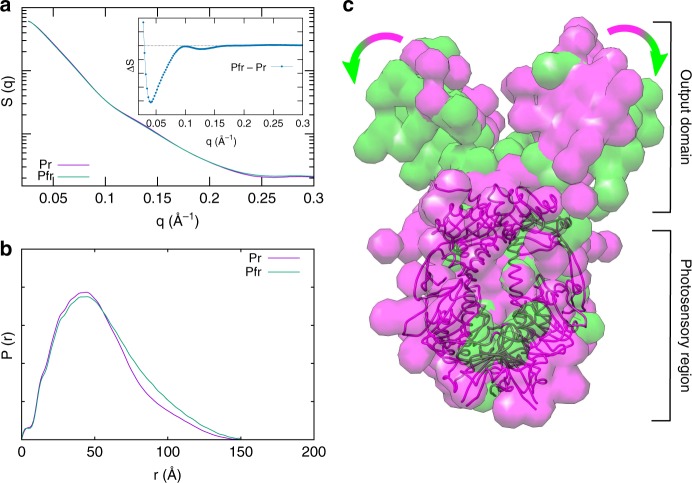
Low-resolution modeling of static X-ray scattering. **a** X-ray scattering patterns of full-length Cph1 in the Pr (pink line) and Pfr (green line) state and their difference (inset). **b** Particle distance distribution function P(r) obtained from scattering patterns. **c** Shape reconstructions with the ab initio models generated by Dammin for full-length Cph1 in Pr (pink envelope) and Pfr (green envelope) state, superimposed to the crystal structure (pdb entry: 2VEA^16^) of the photosensory region of Cph1 in the Pr state (pink sticks)

In order to interpret the difference in the scattering patterns in the small-angle region between the photosensory region and the full-length Cph1 protein we used the absolute X-ray scattering patterns in the q region 0–0.3 Å^−1^ of the full-length Cph1 in both the Pr and Pfr states (Fig. 8a). The difference in the scattering pattern again shows significant differences between the Pr and Pfr states in the small-angle region (Fig. 8a). The particle distance distribution function P(r) obtained from the scattering patterns (Fig. 8b) shows an increase of intensity at high distances, thus revealing an overall expansion of the protein upon the Pr-to-Prf transition. To gain more detailed structural insight and identify the structural domains we performed a low-resolution ab initio modeling of the absolute static patterns of the Pr and Pfr states of full-length Cph1. The models were generated by DAMMIN under the two different illumination conditions and were superimposed onto the structure of the photosensory region of Cph1 in the Pr state^16^. The models show that the dimeric structure is retained in both states and suggest that there is an opening motion of the output domains upon photoconversion (Fig. 8c). As indicated by the arrows in Fig. 8c, the conversion from the Pr state (pink surface) to the Pfr state (green surface) is characterized by an increase of the distance between the output domains. Differences in the photosensory region (which is compatible with the expected overall space distribution) are minor and anyway less evident to interpret.

## Discussion

Members of the phytochrome family share a common photochemical mechanism as the basis of light-signaling, which involves photoisomerization of the bilin chromophore followed by global rearrangement of the protein structure. Until recently the nature of any structural changes in the protein was unknown. The recent X-ray solution scattering measurements on a phytochrome from *Deinococcus radiodurans* have provided a proposed mechanism for transmitting the structural transitions to the kinase output domain to initiate the photoresponse^8,9,11,17^. A change in secondary structure of a unique tongue region is thought to result in the opening of neighboring PHY domains^9^ and in the twisting of the output domains prior to the formation of the final Pfr state^8^. However, it is still unclear whether these structural changes are specific for the bacterial phytochromes and if more substantial structural changes occur in other phytochrome proteins. The results of the present study suggest that the photoinduced Pr → Pfr conversion in the cyanobacterial Cph1 phytochrome from *Synechocystis* sp. PCC6803 involves both localized and global motions on the microsecond-to-second timescale (Fig. 3) and is characterized by more complex structural dynamics than those observed in bacterial phytochromes, where the structural activation of the entire phytochrome occurs in one concerted rearrangement within few milliseconds^8^.

The initial changes at the chromophore of our cyanobacterial Cph1 phytochrome, determined from time-resolved spectroscopic measurements on timescales up to the tens of milliseconds, are unaffected by the presence of the output domain with similar lifetimes of ~ 0.3 ms and ~15 ms for both the photosensory region and the full-length protein. On this same timeframe the time evolution of the photosensory region, derived from the time-resolved X-ray scattering measurements, exactly mirrors the time evolution of the chromophore with a first time constant of also ~0.3 ms. Although this lifetime increases slightly to ~2 ms for the full-length protein, suggesting a minor effect of the output domain on the propagation of the signal from the chromophore to the protein, the shape of the X-ray difference signal at this stage is essentially the same for both forms of the protein. Moreover, the main features observed in the wide-angle region, which reflect more localized structural changes, also appear to be unaffected by the output domain, in both size and time evolution. Even if an univocal connection between X-ray difference signals and structural changes cannot be established for molecules with different resting structures, taken together, these findings suggest that the presence of the output domain in the biologically active full-length Cph1 protein does not significantly alter the nature of the structural perturbation transmitted from the chromophore to the photosensory region of the protein, which has not yet reached the output domain on the millisecond timescale.

On longer timescales considerable differences start to appear in the shape of the X-ray scattering difference patterns between the photosensory region and the full-length protein. Indeed, a large X-ray scattering change is evident in the small-angle region for the full-length protein, indicating that large scale motions occur and most likely involve the transmission of the structural signal to the output domain. Interestingly, both the X-ray scattering and optical spectroscopy kinetics are slightly faster in the full-length protein than in the photosensory domain. This may be due to differences in the solvent environment which may affect the internal protein dynamics and/or to a more efficient dissipation mechanism in the full-length protein where the light perturbation is transferred from the photosensory domain to the output domain. The final structural changes in both forms of the protein occur on slower timescales than is observed spectroscopically, as illustrated by the fact that the structural rearrangement is still evolving after the longest X-ray scattering time-delay of 500 ms. Hence, it is likely that these slower conformational changes in the protein do not impact on the environment of the chromophore as it is not reflected in any noticeable absorbance change. This finding is in contrast to previous studies on the full-length bacterial phytochrome where the main structural changes were completed prior to the final observed spectroscopic evolution^8^.

The second and third basis patterns are likely to represent two different structural intermediates that form on the microsecond-to-millisecond timescale and involve changes in both the small-angle and wide-angle regions. These two basis patterns are essentially identical between the two different forms of the protein. Moreover, the third basis pattern closely resembles the X-ray difference pattern measured by Westenhoff and co-workers in the photosensory domain of the bacterial phytochrome from *Deinococcus radiodurans*, which was attributed to an opening motion of the PHY domains^8,9^. Our findings reveal that this opening motion, which was identified as the final structural event in the Pr → Pfr conversion of the photosensory module of the bacterial phytochrome, is only a transient structural change during the course of the photoinduced structural evolution in cyanobacterial Cph1 phytochrome, as was also recently observed in the photosensory region of an algal phytochrome from *Dolihomastix tenuilepis*^18^. In addition, the same differential signal is present in the full-length form of the protein.

*Ab initio* modeling of the small-angle static patterns of the Pr and Pfr states of the full-length Cph1 protein revealed the nature of the major conformational changes observed (see Fig. 8). The analysis suggested that the main structural event compatible with the X-ray signal change is an opening motion of a part of the dimer interface in the output domain. This structural change is in qualitative agreement with the results previously obtained by means of pulsed electron-electron double resonance (PELDOR) spectroscopy^7^ and is reminiscent of the motions observed in the full-length form of the bacterial phytochrome from *Deinococcus radiodurans*^8^. However, it is important to note that the kinetics of the structural transitions described here for the full-length cyanobacterial phytochrome are considerably more complex than for the bacterial protein. In the case of the bacterial homolog, the structural changes of the entire phytochrome, involving alterations to the PHY tongue region and a rotation of the output domain, occurred in a single concerted rearrangement^8^, complete within a few milliseconds. Conversely, for the cyanobacterial phytochrome we observe multiple structural transitions over the entire µs–s timescale, which are likely to involve similar changes to the tongue region and opening of the neighboring PHY domains, and then leading to the opening motion of the output domains. Our findings suggest that the phytochrome family is characterized by a variety of kinetic pathways that connect the Pr and Pfr states.

The results of the present study will also have implications in the design of optogenetic tools based on phytochromes, where one of the most promising strategies is to replace the natural output domains with other domains that have the desired biological function^3,19,20^. Previous structure-guided design of novel fusion proteins, where the photosensory region of a phytochrome has been linked to a guanylate cyclase enzyme, has highlighted the importance of the linker region in the transmitting the structural changes to the output domain^19,20^. Indeed, our results suggest that the structural dynamics of both the chromophore (time-resolved spectroscopy) and the photosensory region (time-resolved S/WAXS) are largely unaffected by the presence of the output domain up to the millisecond timescale. This separation between the photosensory region dynamics and the output domain on the µs/ms timescale indicates that in this time window the photoreaction can be characterized independently of the nature of the engineered output domain. It is only on slower timescales that the structural changes are propagated to the output domain in a process that is likely to be transmitted through the linker region^19,20^.

## Methods

### Sample preparation

All chemicals were obtained from Sigma-Aldrich unless otherwise stated. The photosensory region (residues 1–514) and full-length Cph1 proteins from *Synechocystis* sp. PCC6803 were produced and purified as a phycocyanobilin-bound holoprotein using a dual-plasmid *Escherichia coli* expression system^7^. The Cph1 genes were synthesized (GenScript) and cloned into the pET21a expression vector (Novagen). BL21 cells containing the two plasmids were grown in 500 ml of Luria–Bertani medium to an OD_600_ of ~0.5 at 20 °C and expression of the genes was induced overnight after addition of 100 µM isopropyl β-D-thiogalactoside. The C-terminal His-tagged proteins were purified as described previously^7^.

### Time-resolved X-ray scattering

TR-S/WAXS experiments have been performed at the beamline ID09 of the European Synchrotron Radiation Facility (ESRF) in Grenoble, France. The protein samples (400 µM) were photolyzed with a circularly-polarized laser pulse (630 nm, 0.5 mJ) from a nanosecond laser (5 ns, EKSPLA) focused with cylindrical lenses to an elliptical spot approximately 2.0 × 0.25 mm^2^ (full width at half-maximum, FWHM) corresponding to a power density of ~1 mJ/mm^2^. To maximize the overlap between the pump- and probe-illuminated volumes, orthogonal pump–probe geometry was employed (Fig. 1). The X-ray beam consisted of pulses of 100 ps (FWHM) in length. The center of the X-ray beam (0.06 × 0.1 mm^2^ FWHM) penetrated a 2-mm-diameter quartz capillary, 0.3 mm from its edge. In order minimize any X-ray radiation damage over a large sample volume and to recover the Pr state of the protein after the laser pump pulse the sample was continuously pumped (Gilson Minipulse 3) from the capillary to a ~1 ml reservoir under 735 nm LED constant illumination (ThorLabs) and then again to the capillary through a teflon loop tubing. The flow speed was set according to both the laser pump–X-ray probe time delay and the repetition rate in order to keep the sample in the pump–probe intersection area during a pump–probe sequence and also to refresh the sample between two consecutive pump pulses. A variable number of single X-ray pulses were selected from the synchrotron pulse train by means of a high-speed chopper and a millisecond shutter. The X-ray pulses scattered by the sample were collected with a 2D detector (Rayonix MX170-HS). Up to 25 patterns per time delay were acquired and averaged together to improve the signal-to-noise ratio. Laser-off images were also acquired with the X-ray pulse arriving 1 ms before the laser pulse and used as a reference to compute the TR-S/WAXS difference patterns. Images were azimuthally averaged and the peak of the undulator spectrum (0.8 Å^−1^) was used as the reference wavelength to convert the scattering angle to the momentum transfer q. All radial patterns were normalized to the water peak (2–2.2 Å^−1^) before calculating differences between laser-on and laser-off patterns. We also checked that the time evolution of the solvent heating peak at 1.8 Å^−1^ did not affect the scattering patterns in the q range where protein difference signal was present (0–0.7 Å^−1^). X-ray scattering data have been also collected on the full-length and photosensory region proteins after illumination with constant 625 and 730 nm LED light to produce Pfr and Pr stationary states, respectively.

### Time-resolved absorption spectroscopy

Laser photoexcitation experiments on the photosensory region and full-length forms of Cph1 were carried out after all samples had been pre-illuminated with a 735 nm LED (Thorlabs) to ensure that the protein was in the Pr state prior to laser excitation. Photoconversion to the Pfr state was triggered by laser excitation at 660 nm (~20 mJ per pulse) in a cuvette of 1 cm pathlength. Absorption transients were recorded at 720 nm using an LKS-60 flash photolysis instrument (Applied Photophysics Ltd)^7^. Lifetimes were obtained from the average of at least five time-dependent absorption measurements by fitting to a triple exponential function. Reduced *χ*^2^ was 2 and 1.5 for the photosensory region and the full-length protein, respectively.

### Singular value decomposition analysis

In order to unveil the kinetic structure of the time-dependent evolution of X-ray scattering patterns, singular value decomposition (SVD) analysis has been performed of the X-ray scattering dataset with a custom-made Python-based code. Briefly, the dataset of time-dependent difference patterns is arranged in a *m* × *n* matrix A, with *m* the number of q values and *n* the number of time delays. The SVD algorithm provides the matrices U and V and the vector S, so that A = U × S × V^T^, where the column of matrix U are called left singular vectors, or basis patterns, the rows of V^T^ are called right singular vectors, or amplitude vectors, and the elements of S are called singular values. The basis patterns are ordered following the high-to-low sorting of singular values. The aim of the SVD analysis of kinetic data is to identify a number of time-independent patterns containing all the relevant information out of the random noise and able to reproduce the dataset as a linear combination of such time-independent patterns. Visual inspection of basis patterns, amplitude vectors and singular values suggested that only the first three basis patterns are significant. Autocorrelation analysis of both basis patterns and amplitude vectors^21^ indicated three components containing significant information, with the rest containing essentially only random noise. A linear combination of the first three basis patterns was then used to reconstruct the entire dataset and to interpret and compare the kinetics of the photosensory region and full-length Cph1 protein.

### Modeling of SAXS data

Static X-ray scattering data on the full-length Cph1 in the stationary Pr state was obtained by continuous LED illumination at 730 nm. Continuous LED illumination at 625 nm produced a Pr/Pfr equilibrium with 75% of the proteins being in the Pfr state, as determined by deconvolution of optical spectra. Static X-ray scattering data of the pure Pfr state was obtained by adding the Pr/Pfr–Pr difference pattern properly rescaled to the pattern of the Pr state. The static patterns of Pr and Pfr states were then used to extract structural information on the large scale protein motions responsible for the small-angle X-ray scattering (SAXS) changes. The pair distance distribution functions P(r) was calculated from the absolute scattering curves on the q range 0–0.3 Å^−1^ using the GNOM software of the ATSAS package^22^. Ab initio envelope models of the Pr and Pfr states were derived using the DAMMIN software (ATSAS package^22^). First, 30 models were generated for each scattering curve, using a P2 symmetry on the basis of the known homodimeric structures of Cph1^16^. DAMAVER software (ATSAS package^22^) was then used to remove outliers, align the remaining models and produce an averaged and filtered model and used as an input for a second DAMMIN analysis. The model produced by this second DAMMIN round with a minimized discrepancy from the scattering data was used to reveal the overall conformational motions responsible for the large difference signal in the SAXS region of the full-length Cph1 patterns (see Fig. 2).

## Data Availability

The datasets generated during and/or analysed during the current study are available from the corresponding authors on reasonable request.
